# A narrative review of the prevalence of polycythemia and its associated risk factors in Saudi Arabia

**DOI:** 10.1097/MD.0000000000042959

**Published:** 2025-06-27

**Authors:** Tareg M. Belali

**Affiliations:** a Faculty of Applied Medical Sciences, University of Bisha, Al Nakhil, Bisha, Saudi Arabia.

**Keywords:** blood indices, erythrocytosis, erythropoietin, JAK2 mutation, polycythemia vera

## Abstract

As polycythemia is a worldwide health concern, Saudi Arabia is experiencing a notable increase in the prevalence of such a disorder. Studying the frequency and cause of polycythemia is an essential part of implementing successful medical treatments and implementing policies that are possibly going to control this disease. The current review aims to underline the key findings of the body of literature that has examined the causes, prevalence, and major challenges associated with polycythemia in the country and correlate those findings with studies that have been conducted around the world. A comprehensive review of the literature was conducted to summarize the major findings of different studies that have been conducted in Saudi Arabia regarding different types of polycythemias, their causes, symptoms, and associated risk factors, including genetic mutations. Various studies have reported different causes and symptoms of polycythemia. Specific mutations in Janus kinase 2 gene have been found to increase the risk of developing polycythemia vera, according to studies that investigated the genetic component of the disease. Furthermore, reports of common polycythemias such as smoker’s, neonatal, and post-transplant polycythemias have been made in different parts of Saudi Arabia. Integrating genetic mutations, demographic information, and clinical manifestations, the current review of the literature offers a comprehensive overview of polycythemia in the kingdom. This benefits polycythemia patients as well as the kingdom by enhancing management techniques, therapeutic approaches, genetic counseling, and the integration of molecular-based diagnostic techniques.

## 1. Introduction

**Figure 1.**
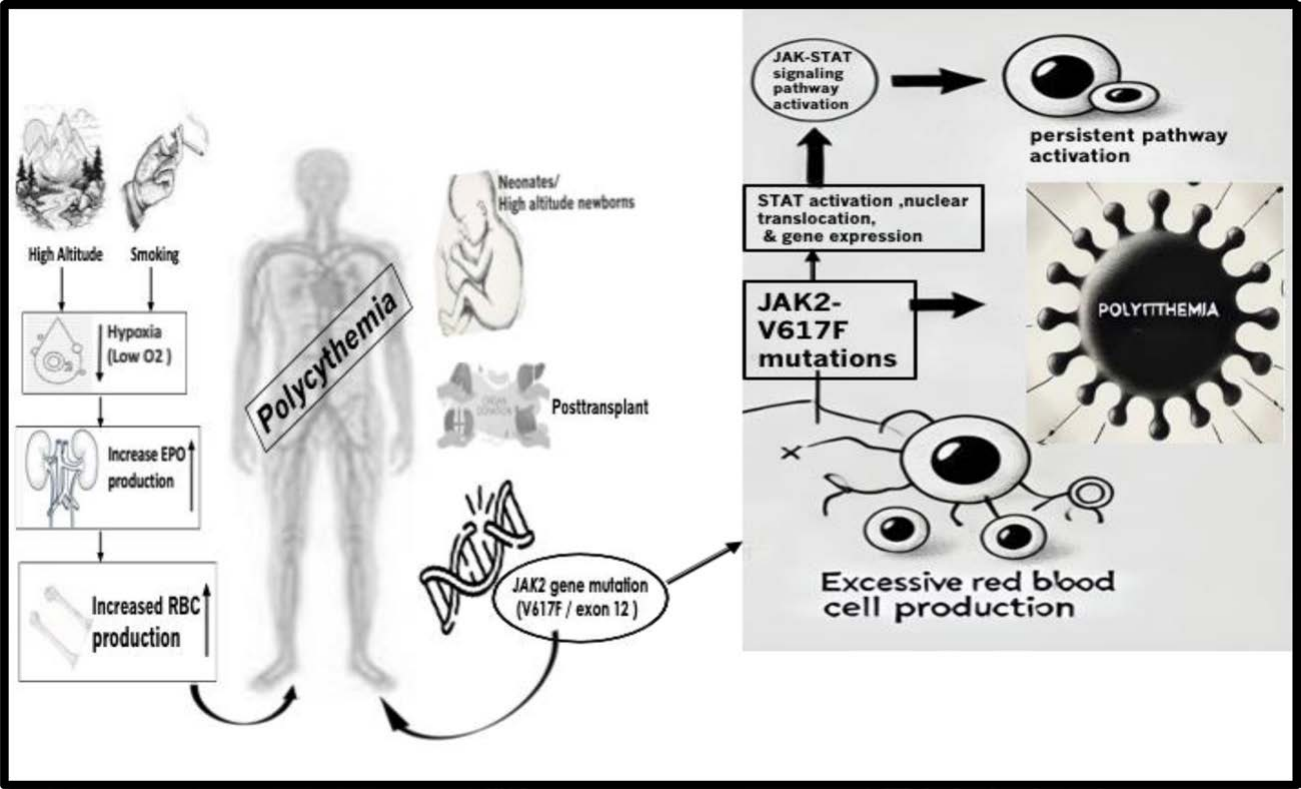
Summary of the causes of polycythemia in Saudi Arabia. High altitude and smoking induce hypoxia that triggers the production of erythropoietin which leads to an increase in RBCs mass. Neonatal polycythemia, neonates born in high-altitude areas, post transplants and genetic mutations of the *JAK2* gene are also among the reported causes of polycythemia among Saudis. JAK-STAT signaling pathway, where cytokine binding activates JAK, leading to STAT activation, nuclear translocation, and gene expression. JAK2 mutations, such as V617F, cause persistent pathway activation, resulting in excessive red blood cell production and increased polycythemia risk. V617F = Valine-to-Phenylalanine Substitution at Codon 617.

The abnormal elevation in hemoglobin (Hb) concentration or hematocrit results in a clinical condition known as polycythemia.^[1]^ In a clinical setting, this elevation is manifested as an increase in the patient’s levels of Hb and hematocrit (HCT). In some cases, this is a physiological change for different age and gender groups.^[2]^ Several pathological, physiological, and environmental factors can result in this clinical condition. Overall, the standardized normal range for healthy adults for Hb is 16 to 18 g/dL while for HCT is 41% to 53%. Factors such as ministration and pregnancy influence the normal ranges for HCT and Hb among females. As for the neonates, they are polycythemic in case the Hb of their central vein is more than 22 g/dL and the HCT is around over 65%.^[3]^ In some cases, high Hb is combined by erythrocytosis which is an increase in the red blood cell mass.^[4]^ At a normal homeostatic condition, the red blood cell (RBC) mass is below 36 mL/kg in men and 32 mL/kg in women. Establishing the normal ranges for blood parameters such as Hb, HCT and red blood cell count depends on the age group, genders, altitude, and ethnicity of any given population.^[5]^ Globally, polycythemia constitutes a major health concern impacting people from different ethnicities, ages, and social statuses. This blood disorder is an intricate challenge for healthcare systems. studying the nature, prevalence, and causes of polycythemia is significant for effective clinical treatment and the planning process of healthcare facilities nationwide. The polycythemia where the Philadelphia chromosome is not detected is a sub-type of polycythemia known as polycythemia vera (PV) which is also a type of myeloproliferative disorder (MF).^[6]^ All 3 major blood components could be overproduced in MF, but an observed increase would be remarkable in the levels of RBC. Multiple studies have been conducted in Saudi Arabia to investigate the causes, risk factors, and distribution of polycythemia. The current publications that studied polycythemia among different Saudi populations who live at different types of topography concluded that living in high altitudes, smoking, post-transplant erythrocytosis, the Janus kinase 2 (JAK2) mutation, and neonatal polycythemia are some of the most reported causes of different types of polycythemias in the kingdom.^[7,8]^ PV, which is caused by an autonomous hyperproduction of red blood cells secondary to myeloproliferative neoplasm, is linked to a genetic mutation in (JAK2). JAK2 mutations, particularly JAK2-Valine-to-Phenylalanine Substitution at Codon 617 (V617F), drive uncontrolled red blood cell production in polycythemia, increasing risks of complications. Monitoring these mutations is vital for diagnosis, risk stratification, and guiding targeted treatments like JAK inhibitors. In Saudi Arabia, access to JAK2 testing is improving in major centers but remains limited in remote areas. Expanding genetic monitoring and integrating it into routine care can enhance personalized medicine, offering targeted therapies and improved disease management. Enhancing diagnostic accessibility and establishing national guidelines for mutation monitoring are crucial to advancing personalized medicine and improving patient outcomes in Saudi Arabia.^[7–10]^ As the hereditary nature among the key causes of familial PV,^[11]^ cultural customs such as consanguinity exacerbate the chance of developing PV. In some Saudi regions, intrafamilial marriage is a common practice resulting in the development of hereditary diseases such as PV and MF.^[9,11]^ The main derivative behind the higher red blood cell count among people who live in high altitudes is the low oxygen (O_2_) concentration that pushes the body to produce more erythropoietin (EPO) and subsequently increases the red blood cell production yielding the physiological polycythemia (Fig. 1).^[12]^ The possible clinical effect of hyperproduction of RBC and platelets because of MF, lies in increasing the risk of developing thrombosis secondary to elevated viscosity of the blood and the higher blood platelet count. Moreover, the deterioration of the clinical presentation of any given PV patient where the condition progresses to leukemia urgently calls for efficient strategic planning to minimize the deterioration of the condition among the Saudi population. Any strategic planning that aims to reduce the frequency of polycythemia among the Saudi population should take into consideration handling specific risk factors. Given the physiological nature behind the development of polycythemia in some cases, the population of high-altitude areas in the kingdom should be educated about the matter and the proper way to handle this condition in case it results in health complications such as high blood pressure. Polycythemia management in Saudi Arabia follows international guidelines, focusing on accurate diagnosis, risk stratification, and treatment to prevent complications like thrombosis. Key strategies include phlebotomy, cytoreductive therapy (e.g., hydroxyurea, interferon-alpha), low-dose aspirin, and JAK2 inhibitors for resistant cases. However, challenges such as limited healthcare access, low public awareness, insufficient data collection, and a lack of localized guidelines hinder effective care. To improve outcomes, investments in healthcare infrastructure, public education, and specialized training are essential, along with the development of national registries and localized clinical protocols.^[12,13]^ Moreover, any effort that aims to minimize the danger of consanguinity should include effective premarital screening that goes together with educating the public about the association between hereditary disorders and consanguinity.^[14]^ Multiple studies have investigated the hereditary factors of PV and have linked specific mutations within the *JAK2* gene to the abnormalities in red blood cell production. for instance, (V617F) mutation within the *JAK2* gene causes a constitutive activation that leads to a simulation of the hematopoietic growth factor function increasing the red blood cell mass.^[15,16]^ Another mutation in exon 12 within the same gene was reported in 2007 and has been linked to the development of PV.^[17]^ Moreover, these mutations have been detected recently within the Saudi population.^[9]^ The recent advancements and development of diagnostic molecular techniques have utilized both V617F and exon 12 mutations in the confirmative diagnosis of PV.^[18]^ This study aims to provide a better understanding about the prevalence of polycythemia and its associated risk factors in Saudi Arabia which will advance strategic planning, medical interventions, resource allocation, and disease management, ultimately improving the quality of healthcare for individuals affected by the condition. To achieve this, after meeting the ethical standards set by Bisha University, the study prioritized selecting articles that specifically address polycythemia’s prevalence and associated risk factors in Saudi Arabia, ensuring relevance, reliability, and cultural context. Recent, high-quality studies with coherent methodologies and accessible data were included, while irrelevant, outdated, or poorly designed studies were excluded. This approach ensures a comprehensive and accurate foundation for guiding healthcare improvements.

## 2. Cigarettes and shisha smoking and the severity of polycythemia

A study that was conducted at King Saud University Medical City in Riyadh associated the severity of polycythemia with cigarette and shisha smoking.^[7]^ In this study, the Hb levels, and the smoking habits of 227 study participants were evaluated. Seventy-six of the subjects were males and 10 were females. 25 of the subjects smoked shisha and 53 were cigarette smokers. Elevated Hb levels (160–168 g/L) were observed in 29.7% of the study participants with 1.5% in both cigarette smokers and shisha smokers, 6% shisha smokers, 37.8% cigarette smokers, and 54.5% nonsmokers (169–171 g/L) which is pre-polycythemic Hb, was identified among 17.6% of the study participants. With 15% shisha smokers, 25% cigarette smokers, 50% nonsmokers, and 10% in both groups. High Hb levels of more than (172 g/L) were polycythemic. Fifty-three percent of the study subjects had >172 g/L Hb levels with 70% smokers with 2% in both cigarette smokers and shisha smokers, 12% in shisha smokers, and 14.8% among cigarette smokers. The average Hb concentration among both cigarette and shisha smokers’ group was 175 g/L, 171.4 g/L for shisha smokers, 170.7 g/L for cigarette smokers, and the nonsmokers had a Hb average of 168.74 g/L. The negative correlation analysis has shown that there was a significant negative correlation (*r* = ‐0.169, *P* = .01) between the smoking habits among the study participants and Hb levels. Study subjects with high abnormal Hb levels smoked nearly 1.6 shisha or 1 cigarette per day. Smokers, to some extent, experience low O_2_ concentration in their blood inducing a cycle of low O_2_-triggering EPO production and then increased RBC production (Fig. 1). Moreover, polycythemic participants had higher shisha and cigarette smoking habits where they smoked 2 shisha and 1.3 cigarettes per day.^[7]^ The effect of smoking cigarettes and shisha on Hb, HCT, RBCs, total white blood cell count, and mean corpuscular hemoglobin concentration (MCHC) in this study resembles that of other studies that have been conducted in Bosnia, Nepal, and England. For instance, a study that has been conducted in Bosnia (Shah et al) where they have described a significant elevation among cigarette and shisha smokers in the levels of Hb, HCT, RBCs, and MCHC.^[19,20]^ In Bosnia, the blood count of smokers has shown a remarkable increase in WBC, Hb, mean corpuscular volume (MCV), and MCHC. This change has impacted males more than females. However, other blood components did not show significant variations.^[21]^ Based on the number of cigarettes smoked each day, a study that was conducted (Whitehead et al) in England found an elevation in all blood cell count as well as HCT, Hb, MCV, and MCHC. However, this study did not find a significant correlation between the amount of cigarette consumption each day and the amount of variation in different blood parameters.^[22,23]^ It is evident from previous studies that the increase of Carboxy Hb among cigarette and shisha smokers leads to the development of polycythemia through an increase of Hb levels secondary to acquired hypoxemia.^[24]^ Moreover, this study conducted in India by Khan et al described how shisha smokers were presented with higher levels of Hb compared to nonsmokers. Nevertheless, both shisha and cigarette smokers have the highest Hb concentration among all groups.^[25,26]^

## 3. Smoking tobacco and secondary polycythemia

A study in Taif City, Saudi Arabia found that heavy tobacco smoking leads to secondary polycythemia and mild leukocytosis.^[27]^ 40 healthy individuals were compared to 40 tobacco smokers in this study. The findings have revealed that a marked increase in Hb, hematocrit, red blood cell, and neutrophil counts, along with overall elevation in platelets and white blood cell counts occurs significantly among tobacco smoking groups compared to nonsmokers. The opposite effect was observed in other factors that are important for blood production. Smoking tobacco has lowered the levels of erythropoietic cytokines such as interleukin-7 (IL-7) and EPO. Moreover, smoking tobacco has influenced some of the genes associated with the regulation of the production of blood cells. This study has observed an upregulation in recombination activating gene 1&2 (RAG-1) and (RAG-2) genes. Additionally, an overexpression of erythropoietin receptor-1 gene was also observed in this study.^[28]^ as a previous study has concluded that tobacco smoking causes systemic hypoxia,^[29]^ this study has described how hypoxia developed among its tobacco-smoking participants. This fact has been also described by Sagone Jr. et al who have observed a reduction in the tissue O_2_ supply among tobacco smokers. They have also reported what has been described before in this review in terms of the significant increase in blood cell count and HCT, Hb MCV, and MCHC among smokers. this study has reported similar observations as well. these findings are indicative of the hyperactivation of the bone marrow activity to bypass smoking-induced tissue O_2_ reduction. negative feedback that results from the increase of Hb and RBCs, prompts the kidney to secrete more EPO. several studies have reported low serum levels of EPO among smokers as the demand for this cytokine rises to stimulate the production of red blood cells.^[30–33]^ this study has reported a molecular mechanism that explains this significant reduction of EPO levels in the serums of smokers. this study found that the receptors of EPO’s mRNA are overexposed leading to an increase of the circulating RBC and Hb levels among smokers. more studies have interpreted this as possible positive feedback that results from the reduced EPO levels of the low serum EPO.^[34–36]^ Following the analysis of whole blood, this study has made a comparison between both groups of participants in terms of levels of white blood cell count. They have found a marked elevation of the WBCs especially the number of circulating neutrophils. A similar finding has been described by previous studies where they revealed that the constant tobacco smoking has caused vascular injury which induces an inflammatory response. This usually leads to a significant increase in different WBCs, including neutrophils, and explains the marked increase of WBCs among the participants of this reviewed study.^[35,37–39]^ Among the findings of this study, a marked increase in the expression of the mRNA coding RAG-1 and RAG-2 genes among smokers. Nevertheless, IL-7 reduces the expression of RAG-1 and RAG-2 genes.^[40]^ A low level of IL-7 among smokers has been detected in this study which explains the main derivative behind the increase of these 2 genes.

## 4. Polycythemic smokers and cluster of differentiation 47 (CD47) downregulation

A cross-sectional study was conducted in the city of Al-Kharj that involved 72 smokers and 50 nonsmokers, along with additional nonsmokers who hadn’t been regularly exposed to smoking and served as controls.^[41]^ Eleven participants were excluded from this study to focus only on genetic problems. Those removed participants have suffered from clinical conditions such as metabolic and immune disorders and other diseases that might serve as interference factors that would be confused with genetic problems. The 61 subjects who were included in this study suffered from other blood disorders such as iron deficiency anemia and polycythemia. The socio-demographic data showed the median age of the study participants was between 15 and 65, 96.7% of the study subjects were men, 65.59% were employed, 73.8% of them were university-educated, and 63.9% were unmarried. There was a variation in the smoking habits of the study participants where 68.9% of the subjects smoked only cigarettes, 14.8% smoked both cigarettes and Shisha, while 16.4% smoked cigarettes and electronic cigarettes, consuming 1 to 20 cigarettes per day. CD47 marker screening by Flow cytometry showed CD47 marker clarity across smoking patterns while correlation analysis detected a significant downregulation in CD47 markers in all types of smokers, especially in cigarette smokers. The variation in the expression of the CD47 marker was also observed among smokers with non-hereditary blood diseases such as iron deficiency anemia and polycythemia. The findings of this study highlight a significant decrease in the expression of CD47 marker on the red blood cell surface in all smokers in comparison to other control groups, despite the type of smoking habit the subjects are accustomed to. The binding of CD47 to thrombospondin-1 and recognition by Signal Regulatory Protein Alpha for phagocytosis by human red pulp phagocytes was enhanced by smoking-induced oxidative stress which also triggered a conformational change in CD47 characteristics. Diseases such as thalassemia and sickle cell anemia usually trigger an increase in eryptosis susceptibility. Moreover, in this study, smokers with polycythemia and iron deficiency anemia were presented with lower CD47 levels compared to healthy nonsmokers which might also accelerate their apoptosis and the removal from the blood circulation by the splenic macrophages.^[41]^

## 5. Predictive factors of post-transplant erythrocytosis

Clinically, post-transplant erythrocytosis (PET), is defined as a perpetual elevation of Hct level of below 51%, a Hb level of a minimum of 16 g/L, or both, when no additional variables are involved.^[42]^ An early study that was carried out at King Faisal Specialist Hospital and Research Centre, Riyadh, Saudi Arabia investigated the factors predictive of post-transplant erythrocytosis. this study analyzed PTE (post-transplant erythrocytosis) among the recipients of renal transplants.^[8]^ 21.6% of the study participants have shown signs and symptoms of PTE such as headache, dizziness, and fatigue with noticeable variation according to different transplant types. The prevalence of PTE was higher among the study subjects who were going through double therapy compared to patients on triple therapy. However, the intensity of the symptoms among the study subjects is often relieved by phlebotomy. Both control groups as well as PTE have shown a tendency to develop Thrombosis to some extent. However, the difference between the 2 groups was not statistically significant. Regardless of whether the study participants have received phlebotomies, Hematocrit levels were elevated. PTE was also reported among some study subjects with no significant change in EPO levels compared to control groups. The study participants with PTE had no remarkable pulmonary complications. Moreover, other factors such as immunosuppressive therapy and creatinine levels have correlated with the occurrence of PTE among participants.^[8]^ The exact cause of PET is yet to be discovered. Preliminary research has found that the production of EPO from a functioning allograft starts in 2 to 4 2 weeks after the PTE incident. This type of temporary transient PTE occurs among transplant patients possibly because of The gradual decrease in EPO levels over time.^[43]^ This study has associated 3 factors that could predict the risk of developing PTE. These factors include the levels of serum creatinine at the beginning of PET, the type of immunosuppression used in treatment, and the duration of dialysis. the level of serum creatinine could be indicative of the impaired production of red blood cells due to the slow function of allograft which makes it difficult to correct the anemia.^[44]^ This study has also shown that patients had low levels of serum creatinine at the beginning. This highlights the good performance of the allograft in RBC production. Moreover, the allograft is more likely to be the main source of EPO, however, the role of the original kidneys cannot be excluded.^[45,46]^

## 6. Neonatal polycythemia in Jeddah

Another study evaluated the risk factors, symptoms, pattern, and management of polycythemia at the Neonatal Intensive Care Unit (NICU) at Al Aziziah Maternity and Children Hospital in Jeddah.^[10]^ At the NICU, the prevalence of polycythemia among 101 neonates was 14.5% with a mean gestational age of 38.1 weeks. Most of them were full-term infants while 22 were preterm babies with most male infants delivered by cesarean section. Furthermore, this study has evaluated different risk factors that could lead to the increase of polycythemia prevalence among the study subjects. For instance, risk factors such as maternal pregnancy-induced hypertension, small for gestational age, and infants of diabetic mothers may increase the incidence of polycythemia among newborns. Moreover, symptoms such as thrombocytopenia, jaundice, lethargy, hypocalcemia, poor feeding, tachypnea, and hypoglycemia were observed among polycythemic infants. Approximately 40% of the neonates have shown no symptoms while 17% were incidentally discovered during medical examination. The length of stay at the NICU was significantly influenced by factors such as having oliguria and Tachypnea. high hematocrit levels among participants were treated by conservative treatment, IV fluid boluses, and partial exchange transfusion intravenous fluid transfusion interventions. Nevertheless, those interventions have shown varying success rates as the hematocrit average was lowered to nearly 2.8 hours after treatment and maintained to gradually decrease afterward.^[10]^

## 7. Incidence of polycythemia in high altitude versus sea level cities

Alkhaldy et al have investigated the effect of altitude on Hb and red blood cell indices in adults in different regions of Saudi Arabia. this study examined over 120,000 participants who live in different cities located in the mountains as well as coastal cities.^[47]^ Following screening the participants, the subjects were narrowed down to 46,012 from the coastal city of Jeddah, 5216 from Taif, and 5831 from Abha, both are high-altitude cities. Analyzing the Hb concentrations of the study participants has shown altitude-dependent variation among the subjects. For instance, participants from Abha and Taif were presented with higher Hb concentrations compared to sea level Jeddah. There was a 97.5% higher Hb concentration among the participants from high altitudes where women had a greater significant increase in Hb compared to men. Variations in blood indices as well as red blood cell count and Hb concentration were significant between genders and across different cities. This study observed that the MCV values were lower in participants from Abha compared to the other subjects from Taif and Jeddah. However, no significant differences in Hb levels among different age groups (young and middle-aged) were observed in the study.^[47]^

## 8. High-altitude and polycythemic newborns

Another study assessed the values of Hb and hematocrit among Saudi neonates born in the high altitude of Abha and compared these values with known values of other lowland areas of Saudi Arabia. This study included 587 infants who were born in 1993.^[48]^ Analyzing maternal venous blood components showed an average Hb of 122 ± 11 g/L and mean hematocrit of 0.347 ± 0.028 L/L, not influenced by the age and similarities of the mothers. Investigating the parameters of cord blood among the 587 infants showed Hb values of 140 and 210 g/L in 90.3% of the subjects. Hematocrit on the other hand was higher than 210 g/L in 8.6% of the infants. For hematocrit, 82.1% fell between 0.42 and 0.63 L/L, and 16.6% were polycythemic newborns. Compared to those in Riyadh and Jeddah, Abha Newborns had significantly higher red cell values (*P* < .001), this might be due to the hypoxia induced by the high altitude of Abha city.^[48]^

## 9. Post-transplant erythrocytosis

A study that was conducted on the renal transplant recipients at Jeddah Kidney Center investigated post-transplant erythrocytosis among patients.^[49]^ This study investigated 1655 patients who received transplants at the center. Among the study participants, 9.6% developed post-transplant erythrocytosis (PTE), where males were affected by the condition more than females (154 men, 5 women) with a mean age of 42 ± 9 years. Different renal clinical conditions were also observed among participants who received immunosuppressive regimens such as azathioprine, prednisolone, and cyclosporine. typically, patients develop PTE around 8.2 ± 5 months after the transplant. This type of erythrocytosis usually lasts around 10.3 ± 3 months. Recovery has been reported in all study participants after receiving different treatments such as angiotensin-converting enzyme inhibitors or phlebotomy. 34.6% of the patients have suffered from hypertension however this condition was well-controlled. 5.6% of study subjects were presented with chronic allograft nephropathy, however, most of these patients maintained normal function of their renal grafts. Overall, the participants of this study have experienced a benign form of PTE, that was more prevalent in males than females. PTE appeared among the study participants within the first year after receiving the transplants. Nevertheless, this study did not report any adverse impact on the renal graft functions among its subjects.^[49]^

## 10. JAK2 mutation and PV

At King Faisal Specialist Hospital and Research Centre in Riyadh, Alghasham et al used Sanger sequencing to identify mutations in JAK2 exons 12 to 15 among patients harboring mutations linked to PV, essential thrombocythemia and MF.^[9]^ Initially, this study involved 1811 samples, but 1706 samples were analyzed for JAK2 mutations as 105 of the samples were deemed duplicates or insufficient. JAK2 mutations were detected in 16% of the samples where 123 were male subjects and 148 were females. The median age of the subjects was 54 years. However, there was a single pediatric case a 4-month-old diagnosed with MF. Among the 271 subjects with JAK2 mutations, 96.7% expressed p.V617F mutation. Other mutations were detected among the 3.3% of the study group including mutations in exon 13 and exon 12 deletions. Five percent of patients of the study participants were presented with other genetic abnormalities such as methylenetetrahydrofolate reductase gene polymorphisms and abnormal karyotypes. A minor fraction of the study participants, (1.1%), were harboring both p.V617F and the Breakpoint Cluster Region/Abelson Murine Leukemia Viral Oncogene Homolog 1 fusion gene. Eighty-seven of the patients were diagnosed with myeloproliferative neoplasms with 35 with PV, 22 with essential thrombocythemia, and 11 with MF. PV patients with exon 12 JAK2 mutation have isolated erythrocytosis at clinical presentation.^[9,50]^

## 11. Conclusions

Studies on polycythemia in Saudi Arabia offer useful understandings of the frequency, risk factors, and the proper clinical intervention. Hereditary aspects, especially the consequences of intrafamilial marriages, contribute to the spread of PV. Distinctive mutations in the *JAK2* gene are associated with the risk of developing PV and MF. The physiological aspect of polycythemia development among the high-altitude population is emphasized by epidemiological research conducted within topographies. Smoking has also been identified as a major risk factor for polycythemia as it induces hypoxia and downregulates the levels of CD47. Post-transplant erythrocytosis and neonatal polycythemia were also reported among Saudis (Fig. 1). To manage polycythemia, comprehensive screening programs, especially with modern molecular genetics, and raising awareness are essential. Effective prevention and management plans that take into consideration different geo-cultural factors are also important. Additionally, the recent development in molecular techniques enhances the screening for different genetic mutations linked to the development of polycythemia. Identifying these genetic defects will help understand the hereditary nature of the disease and will help in the development of personalized medical care.

## Acknowledgments

The author is thankful to the Deanship of Graduate Studies and Scientific Research at the University of Bisha for supporting this work through the Fast-Track Research Support Program.

## Author contributions

**Conceptualization:** Tareg M. Belali.

**Data curation:** Tareg M. Belali.

**Writing – review & editing:** Tareg M. Belali.
